# A retrospective report on the preoperative mandatory SARS-COV-2 infection screening in a single pediatric center. Is it time to stop testing our patients?

**DOI:** 10.1590/0100-6991e-20223433-en

**Published:** 2022-12-09

**Authors:** CEZAR DANIEL SNAK DE SOUZA, TATIANA SARUHASHI, MARIANA FONTES NEVILLE LIMA, FRANCISCO IVANILDO DE OLIVEIRA, DEBORA DE OLIVEIRA CUMINO

**Affiliations:** 1 - Hospital Infantil Sabará, Anestesiologia - São Paulo - SP - Brasil; 2 - Hospital Infantil Sabará, Centro de Controle e Infecção Hospitalar - São Paulo - SP - Brasil

**Keywords:** COVID-19 Testing, Perioperative Period, Anesthesia, General Surgery, Teste para COVID-19, Período Perioperatório, Anestesia, Cirurgia Geral

## Abstract

The novel coronavirus SARS-COV-2 (COVID-19) pandemic dramatically changed the workflow of healthcare professionals around the world. Surgical procedures were withheld and postponed in a scenario of fear and uncertainty. Despite numerous medical institutions having swiftly and widely implemented pre-operative screening protocols, cost-effective studies remain scarce specially when comparing to other mitigation measures such as the donning of masks and social distancing measures. The objective of our study is to report the monthly positivity rates of SARS-COV-2 infection in our service and compare our data with monthly positivity rates reported by the State Health Department. Between April, 2020, to February, 2022, 7,199 patients had the RT-PCR for SARS-COV-2 collected, with 187 (2.59%) testing positive for COVID-19. Most of them (62.1%) were asymptomatic. The most common symptoms were coryza (10.7%), fever (10%), and diarrhea (8.7%). Nonetheless, there were two deaths due to COVID-19 reported in our center. Further studies are necessary to elucidate the impact of pre-operative screening for SARS-COV-2 in asymptomatic patients.

## RESUMO

A pandemia causada pelo novo coronavírus (SARS-COV-2) alterou a rotina de pacientes, profissionais e serviços de saúde em todo o mundo levando ao adiamento ou suspensão de muitos procedimentos cirúrgicos. Muitos serviços implementaram protocolos de triagem sistemática pré-operatória para SARS-COV-2. No entanto, segue incerta a eficácia da triagem sistemática de pacientes assintomáticos para SARS-COV-2 quando comparada com outras medidas de mitigação como o uso de máscaras e o distanciamento social apesar da aparente sensação de segurança conferida pela testagem pré-operatória de pacientes para SARS-COV-2. Sendo assim, descrevemos a experiência de dois anos de um hospital pediátrico (n=7.199) na triagem sistemática pré-operatória para SARS-COV-2 e comparamos a taxa de positividade mensal reportada pelo serviço em relação a taxa de positividade mensal regional do Estado de São Paulo. No período de Abril de 2020 a Fevereiro de 2022 houveram 187 (2,56%) RT-PCR positivos para COVID-19 dos quais 62.1% assintomáticos. Os sintomas mais comuns foram coriza (10,7%), febre (10%) e diarreia (8,7%). Dois pacientes faleceram em decorrência de sintomas relacionados a COVID-19. Mais estudos são necessários para determinar o impacto da testagem pré-operatória de RT-PCR para SARS-COV-2 em pacientes assintomáticos na mitigação da transmissão intra-hospitalar e na morbidade cirúrgica.

In early 2020, the routine of surgical centers was highly impacted by the pandemic and, in a scenario of fear and uncertainty, many elective surgeries were postponed or suspended. As of May 2020, studies on patients undergoing surgery with SARS-COV-2 infection already showed a 23.8% increase in mortality and a 53.2% increase in pulmonary complications^1^, and operating room professionals were concerned about the risk of intra-hospital transmission of the virus at a time when vaccines were not yet available^2^. Although many of these series included pediatric patients, we still do not know the impact of COVID-19 on the outcome of pediatric surgical patients. In light of current data, one could speculate that the risk of complications is lower than in adults, either because of the milder presentation of the disease or by the profile of surgeries, which are often shorter and less invasive^3^.

The institution of preoperative systematic screening protocols for SARS-COV-2 mitigated this situation, by potentially reducing the risk of adverse effects in patients and minimizing occupational exposure in health professionals. In addition, it avoided spending on protective materials in cases of uncertain diagnosis, strengthening hospital quality and safety processes^4^. Although adherence by hospitals in Brazil has been wide, studies regarding its effectiveness and cost-effectiveness remain scarce^5^. In the pediatric population - relatively spared from severe forms of the disease -, the lack of evidence is even more critical.

The hospital infection control service, with the aim of reducing the risk of intra-hospital transmission of COVID-19, has established the mandatory use of N95/PFF2 masks for all employees since April 2020. In addition, it has established epidemiological and symptoms screening, with application of a structured questionnaire in the pre-anesthetic evaluation, and collection of RT-PCR for SARS-COV-2 up to 72 hours before the procedure. Elective cases with positive RT-PCR were postponed for at least 15 days from the examination date. In face of moderate to severe clinical conditions, or in immunosuppressed patients, surgery was postponed for at least 20 days. Children with negative RT-PCR, with mild respiratory or flu symptoms, could be operated on after symptoms improvement if the exam had taken place 24 hours after symptoms onset.

RT-PCR for SARS-COV-2 has been collected systematically since April 2020. Therefore, the objective of the present study is to describe the positivity rates in one of the largest private pediatric hospital centers in Brazil and, due to the ubiquity of the spread of SARS-COV-2 during the pandemic period, compare them with the general positivity rates of the population of the State of São Paulo, from April 2020 to February 2022, available on a freely accessible digital platform by the State Department of Health^6^.

During the study period, 7,199 patients underwent preoperative RT-PCR for SARS-COV-2, and 187 (2.59%) cases of COVID-19 were diagnosed, most of which (62.1%) were asymptomatic (Table 1).

**Table 1:** Preoperative patient data.

Collection time	04/01/2020 to 02/28/2022
Patients with collected RT-PCR	7,199
Preoperative positive RT-PCR	187 (2.59%)
Reassessed patients	177 (94.65%)
Days elapsed until evaluation	3 (2-22)
Sex	138M 49F
Age (months)	03.44 (23.51-79.55)
Presence of Comorbidities ( ASA >1)	101 (57.0%)
Reported Symptoms	
Asymptomatic	110 (62.1%)
Fever	15 (8.4%)
Cough	13 (7.3%)
Dyspnea	4 (2.2%)
Runny nose	16 (9.0%)
Diarrhea	13 (7.9%)
Decline in general condition	8 (4.5%)

Note: Categorical variables expressed as proportions and continuous variables as median (IQR 25-75).

**Figure 1:**
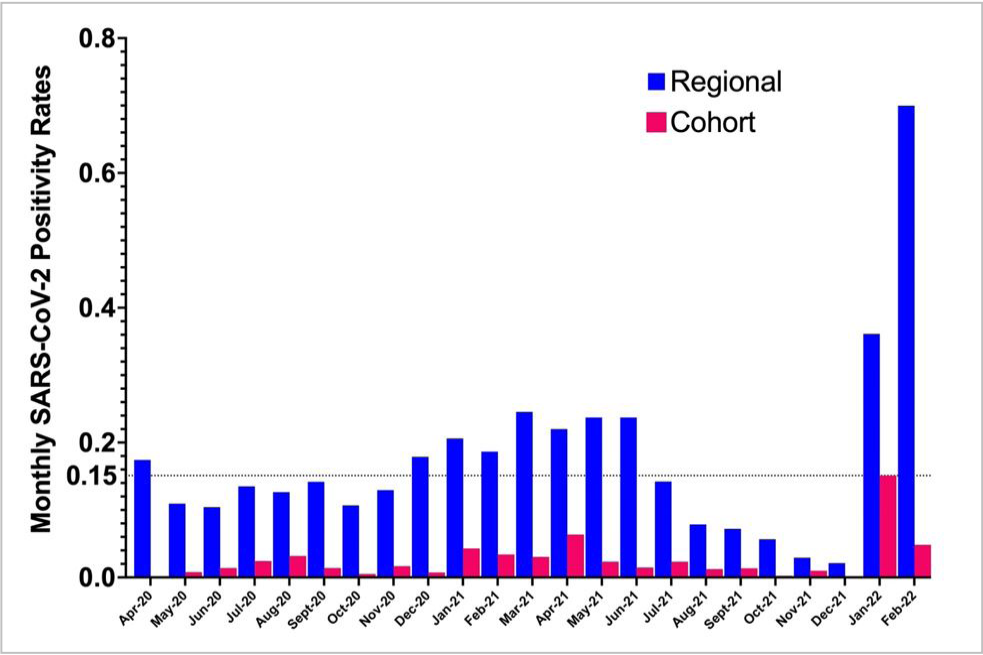
Positivity rate in the General population of the State of São Paulo x Surgical patients at the institution.

The comparison of the monthly positivity rate in our service with the general positivity rate in the State of São Paulo can be seen in Figure 1, and the clinical and demographic characteristics of the patients, in Table 1. The average monthly positivity rate was 2.56 % (95% CI 1.19-3.93%) compared with the overall rate for the State of São Paulo of 17.4% (95% CI 11.37-23.41%, p<0.001). The symptoms presented by the positive patients are described in Table 1, with fever, runny nose, cough, and diarrhea being the most commonly reported. Nielson et al. reported a similar pattern of symptoms, the most common being fever, fatigue, nausea, and vomiting, as well as an increase in pulmonary complications^7^. In our service, two patients (1.06%) had a worsening condition requiring hospitalization. The first patient had Becker Muscular Dystrophy due to an unspecific increase in hepatic transaminases and the second patient was dependent on mechanical ventilation due to a worsening of the breathing pattern. However, two patients died as a result of the diagnosis of COVID-19 during the study period.

Our results are in line with other studies published in France^8^ and USA^9^ in pediatric surgical populations that described RT-PCR positivity rates between 0.93% and 1.2% compared with 2.56% reported by our service. As for other regional services, Aguiar et al. reported a positivity rate of 6.8-7.6% in asymptomatic adult surgical oncology patients^10^. In light of current data, one can speculate that the transmission patterns are similar between services around the world.

With the cooling of the pandemic, after the discharge caused by the Ômicron variant, the benefit of performing the preoperative screening test in children can be questioned, considering:

1. The hospital cost of carrying out RT-PCR tests for SARS-COV-2, without reimbursement from health plans;
2. The variable sensitivity of the test in asymptomatic patients, between 70 and 90%;
3. The progressive drop in the number of new cases of COVID-19;
4. The similar probability of post-negative RT-PCR disease in asymptomatic patients when compared with the probability of disease in non-tested patients in a population with low disease prevalence;
5. The strain and stress caused to children and families by the lack of collaboration and difficulty in collecting nasal swabs, with a negative impact on the patient’s experience^5^.

But why, even with little evidence, are systematic screening protocols maintained and little questioned? First, for the sense of security given to patients, family members and health professionals, this variable being difficult to measure objectively. It is a fact that systematic screening increased the feeling of security and contributed to the resumption of elective surgeries, the number of which remained high even in periods of high general incidence of the disease. Second, because children with COVID-19 are mostly asymptomatic or oligosymptomatic. However, with the expansion of vaccine coverage, it is necessary to question for how long preoperative screening will still be necessary and what parameters should be considered as a support metric for maintaining preoperative testing policies.

In our study, we did not analyze the impact of other factors, such as the availability of personal protective equipment (PPE) and dedicated hospital flow for patients with a confirmed diagnosis of COVID-19 to minimize intra-hospital viral transmission. More studies are needed to determine the true impact of preoperative RT-PCR testing for SARS-COV-2 of asymptomatic patients on mitigating in-hospital viral transmission and surgical morbidity.
